# Robotic thymectomy for ocular myasthenia gravis: a case series from a UK tertiary centre

**DOI:** 10.1136/bmjno-2025-001314

**Published:** 2025-12-16

**Authors:** Thomas Roberts, Stephen Cone, Jennifer Spillane, David Lawrence, Alessandro Maraschi, Kunal Bhakhri

**Affiliations:** 1 University College London, London, England, UK; 2 UCL Medical School, London, UK; 3 University College Hospital, London, England, UK; 4 Neurology, Queen Square Centre for Neuromuscular Diseases, London, England, UK; 5 MRC Centre for Neuromuscular Disorders, London, London, UK; 6 University College Hospital at Westmoreland Street, London, England, UK; 7 Thoracic Surgery, University College Hospital at Westmoreland Street, London, England, UK

**Keywords:** MYASTHENIA, NEUROIMMUNOLOGY, NEUROMUSCULAR, SURGERY

## Abstract

**Background:**

Ocular myasthenia gravis (oMG), characterised by ptosis and diplopia, may progress to generalised MG (gMG). Thymectomy is established for seropositive gMG, but its role in oMG remains unclear. Robotic-assisted thoracoscopic surgery (RATS) offers minimally invasive advantages, yet outcomes in oMG are understudied. This case series describes the outcomes of seven patients at University College London Hospitals (UCLH) (2019−2025) undergoing robotic thymectomy for oMG.

**Methods:**

We describe seven oMG patients undergoing robotic thymectomy at UCLH (2019−2025), focusing on improvement in daily life using the MG activities of daily living (MG-ADL) score and medication reduction.

**Results:**

Patients showed significant pyridostigmine reduction (mean decrease: 246 mg) and improved MG-ADL scores (mean=4.00 to 0.57) with no major complications. No patients progressed to gMG.

**Conclusion:**

We saw robotic thymectomy (RATS) reduce pyridostigmine dependence and improve quality of life (MG-ADL) in the seven oMG patients, with no complications. These cases demonstrated successful cases of RATS as a transformative, minimally invasive option for early MG. While thymectomy may reduce the risk of generalisation, further multicentre studies are needed. Careful patient selection remains critical, but RATS may expand feasibility in oMG.

## Introduction

 Myasthenia gravis (MG) is a rare neuromuscular condition affecting approximately 15 per 100 000 individuals in the UK.^1^ An autoimmune disease, MG affects the neuromuscular junction and, as such, manifests as a weakness of voluntary muscles, worsening with repeated or sustained use. Symptoms range from ptosis and diplopia to systemic muscle weakness causing dysarthria, dysphagia and shortness of breath.^1^

MG onset begins with ocular manifestation in 50%–85% of cases,^2^ with both ocular MG (oMG) and generalised MG (gMG) showing high rates of anti-acetylcholine receptor (AchR) antibody positivity.^3^ oMG manifests as a milder phenotype, with symptoms limited to the eyes. Conversion rates to gMG vary widely (between 20%–80%), underscoring its unpredictable course.^4^

The thymus plays a central role in MG pathogenesis, with thymic hyperplasia and thymoma present in 70% and 10%–15% of anti-AchR antibody-positive patients, respectively.^5^ Even in OMG, thymic abnormalities are prevalent, suggesting early thymectomy may mitigate autoantibody production.^6^ Current guidelines recommend thymectomy for thymoma-associated MG and generalised AchR antibody-positive MG,^7^ but its role in oMG remains debated.

While acetylcholinesterase inhibitors and corticosteroids are first-line therapies, pyridostigmine is not always effective and long-term steroid use carries significant morbidity, prompting interest in surgical options.^8^

Minimally invasive techniques, particularly robotic-assisted thoracoscopic surgery (RATS), have surpassed open and VATS approaches in precision and recovery times.^9^ We describe our experience with robotic thymectomy in seven oMG patients, reporting postoperative outcomes, medication use, MG activities of daily living (MG-ADL) scores and progression to generalised disease.

## Methods

### Study design

We retrospectively identified all patients diagnosed with oMG who underwent thymectomy at University College London Hospitals (UCLH) between 2019 and 2025. Patients were referred from the National Hospital for Neurology and Neurosurgery. Demographics, MG diagnosis date, thymectomy date and thymoma presence were recorded.

### Population

Patients were included if they underwent thymectomy for oMG at UCLH (2019–2025). The oMG subgroup required MG-ADL scores reflecting only ocular symptoms, with no prior evidence of generalisation. Exclusions applied if MG management occurred outside UCLH/Moorfields Eye Hospital. Robotic thymectomy eligibility was determined case-by-case in MDT discussions.

### Data collection

Patients were identified via UCLH’s ‘EPIC’ system, with informed consent obtained preoperatively.

## Results

We identified seven patients with oMG who underwent robotic thymectomy at UCLH. Thymectomy was carried out by thoracic surgical consultants with extensive robotic experience. Baseline characteristics are shown in table 1.

**Table 1:** Baseline clinical characteristics of the ocular myasthenia gravis patients

Patient	Gender	Age*	Preoperative MG-ADL	AChR Ab status	Preoperative AChR titre (nmol/L)	Preoperative pyridostigmine (mg/day)	Preoperative prednisolone	Preoperative steroid-sparing agent
1	F	37	5	Positive	10.2	300	No	No
2	M	21	3	Positive	20	540	No	No
3	M	34	6	Positive	20	540	No	No
4	F	36	2	Positive	20	240	No	No
5	F	33	6	Positive	6.27	120	No	No
6	F	65	4	Positive	10.2	0	No	No
7	M	26	2	Not tested	N/A	0	Yes (7.5 mg)	No

* Age calculated from Date of Birth and Date of Thymectomy.

AChR, acetylcholine receptor; MG-ADL, myasthenia gravis activities of daily living.

### Patient characteristics and preoperative status

The series comprised four women (57.1%) and three men, with a mean age of 34.1 years. At presentation, the mean MG-ADL score was 4.00 (±1.73). Most patients (n=6, 85.7%) were AChR antibody-positive, with a mean titre of 14.4 nmol/L (±6.1). Preoperatively, five patients (71.4%) were on pyridostigmine (mean 348 mg/day±186.9). One patient (14.3%) was on low-dose prednisolone (7.5 mg/day). No patients were taking steroid-sparing agents. One patient received intravenous immunoglobulin (IVIG) as a preoperative optimisation measure.

### Surgical and histopathological findings

All seven patients successfully underwent RATS thymectomy. Complete thymic resection was achieved with clear margins, no conversion and minimal blood loss. Histopathology revealed thymic abnormalities in six patients (85.7%): thymic hyperplasia (n=2), follicular hyperplasia (n=2), thymic aplasia/dysplasia (n=1) and one type AB thymoma (n=1). One specimen (14.3%) was histologically normal. These findings are summarised in online supplemental table 1.

### Postoperative clinical course and outcomes

At a mean follow-up of 18.6 months (±15.2), symptom improvement was observed. The mean MG-ADL score decreased from 4.00 to 0.57 (online supplemental table 2). No patient experienced post-operative myasthenic crisis or progression to gMG.

Pyridostigmine use decreased, with the mean daily dose falling from 348 mg to 102 mg (online supplemental table 3). The patient prescribed prednisolone preoperatively discontinued steroid use. However, two other patients started low-dose prednisolone (10 mg/day), and one commenced mycophenolate mofetil (2 g/day) to aid minor ocular symptom increase at the time of follow-up. Despite this, generalised symptoms remained absent.

## Discussion

The management of oMG is primarily medical, but thymectomy is a recognised disease-modifying intervention in AChR antibody-positive disease. While established in gMG following the MGTX trial,^10^ its role in oMG is less defined. This series describes the feasibility and outcomes of RATS thymectomy in seven oMG patients.

Our patients demonstrated marked symptomatic improvement post-thymectomy, with the mean MG-ADL score falling from 4.00 to 0.57. Pyridostigmine requirement was reduced. Critically, no patient progressed to gMG during follow-up. This aligns with studies reporting lower conversion rates post-thymectomy compared with medically managed cohorts.^11 12^ Furthermore, thymectomy for oMG is supported by historical evidence. Schumm *et al* demonstrated that sternotomy-based thymectomy in 18 oMG patients resulted in 80% symptom improvement, a 0% generalisation rate and reduced pyridostigmine use in 89% of patients^13^—resonating with our observed cohort.

A key focus is the RATS technique. We found RATS feasible and well-tolerated, with a mean postoperative stay of 2.3 days and no major complications. This aligns with known advantages of minimally invasive techniques over sternotomy.^14^ Reduced tissue trauma with RATS may mitigate triggers for postoperative exacerbation, contributing to the swift recoveries observed.

Patients were predominantly AChR antibody-positive, reflecting the subgroup most likely considered for thymectomy. Serological heterogeneity underscores the importance of careful patient selection.

## Limitations

The primary limitation is its design as a small, single-centre case series without a control group. The findings are descriptive and hypothesis-generating.

## Conclusion

This case series describes RATS thymectomy in seven oMG patients. In this cohort, RATS was associated with meaningful clinical improvements, including reduced symptom severity and decreased pyridostigmine dependence. No patient progressed to gMG. The RATS procedure demonstrated a compelling safety profile, with minimal blood loss, no major complications and short hospital stays.

Our findings contribute to evidence suggesting a potential role for thymectomy in oMG management, particularly for AChR antibody-positive patients. The minimally invasive nature of RATS appears to mitigate traditional surgical risks, potentially making early intervention more viable.

While promising, these results are limited by the study’s design and sample size. The outcomes underscore the need for larger, prospective studies to definitively establish the efficacy of RATS thymectomy in altering the natural history of oMG. Nevertheless, within a multidisciplinary team, RATS thymectomy can be a safe and potentially transformative intervention for oMG.

## Supplementary material

10.1136/bmjno-2025-001314 online supplemental file 1

## Data Availability

All data relevant to the study are included in the article or uploaded as supplementary information.
