# Supplementary material for: Robotic thymectomy for ocular myasthenia gravis: a case series from a UK tertiary centre
Source: BMJ Neurol Open. 2025 Dec 16;7(2):e001314. doi: 10.1136/bmjno-2025-001314 (PMC12716504; doi:10.1136/bmjno-2025-001314)
Supplement: online supplemental file 1 [file bmjno-7-2-s001.docx]

**Supplemental Table 1: Surgical and Histopathological Details**

| **Pt.** | **Past Medical History** | **Conversion** | **Operative Time (mins)** | **Post-Op Hospital Stay (days)** | **Intra/Post-Op Complications** | **Histopathology** |
| --- | --- | --- | --- | --- | --- | --- |
| 1 | Hypothyroid,  antiphospholipid syndrome | No | 109 | 3 | None | Thymic Aplasia / Dysplasia |
| 2 | Nil | No | 98 | 3 | None | Thymic Follicular Hyperplasia |
| 3 | Asthma, bicupid aortic valve | No | 121 | 2 | None | Thymic Follicular Hyperplasia |
| 4 | Nil | No | 92 | 1 | None | Thymic Hyperplasia |
| 5 | Nil | No | 105 | 2 | None | Normal Thymic Tissue |
| 6 | Asthma | No | 120 | 3 | None | Type AB Thymoma (pT1A) |
| 7 | SVT paroxysmal | No | 99 | 2 | None | True Thymic Hyperplasia |

**Supplemental Table 2: Summary of Descriptive Outcomes for the oMG Case Series (n=7)**

| **Parameter** | **Pre-Operative (Mean ± SD or n)** | **Post-Operative (Mean ± SD or n)** | **Descriptive Change** |
| --- | --- | --- | --- |
| **MG-ADL Score** | 4.00 ± 1.73 | 0.57 ± 0.79 | Decrease |
| **Pyridostigmine Dose (mg/day)** | 248.6 ± 218.4* | 72.9 ± 159.1* | Decrease |
| **Patients on Prednisolone** | 1 (14.3%) | 2 (28.6%) | Slight Increase |
| **Conversion to gMG** | 0 | 0 | None |
| **Operative Time (mins)** | 106.3 ± 11.0 | N/A | - |
| **Hospital Stay (days)** | 2.3 ± 0.8 | N/A | - |

**Supplemental Table 3: Clinical and Pharmacological Outcomes at Follow-up**

| **Pt.** | **Follow-up (Months)*** | **MG-ADL Score** |  | **Pyridostigmine Dose (mg/day)** |  | **Prednisolone Dose (mg/day)** |  | **MMF Dose (g/day)** |  | **Conversion to gMG** |
| --- | --- | --- | --- | --- | --- | --- | --- | --- | --- | --- |
|  |  | Pre-Op | Post-Op | Pre-Op | Post-Op | Pre-Op | Post-Op | Pre-Op | Post-Op |  |
| 1 | 26 | 5 | 1 | 300 | 0 | 0 | 10 | 0 | 0 | No |
| 2 | 47 | 3 | 0 | 540 | 450 | 0 | 0 | 0 | 2 | No |
| 3 | 41 | 6 | 2 | 540 | 30 | 0 | 0 | 0 | 0 | No |
| 4 | 20 | 2 | 0 | 240 | 30 | 0 | 0 | 0 | 0 | No |
| 5 | 55 | 6 | 0 | 120 | 0 | 0 | 10 | 0 | 0 | No |
| 6 | 9 | 4 | 0 | 0 | 0 | 0 | 0 | 0 | 0 | No |
| 7 | 2 | 2 | 1 | 0 | 0 | 7.5 | 0 | 0 | 0 | No |
